# Impact of recyclability on the tensile and Impact properties of coated plastic materials for the automotive and electronic sectors

**DOI:** 10.12688/openreseurope.16888.4

**Published:** 2025-09-09

**Authors:** Vanessa Ventosinos Louzao, Denise García Murias, Miguel Ángel De Dios Álvarez, Pablo Alberto Acuña Domínguez, Esteban Paredes Barros, Raquel Ledo Bañobre

**Affiliations:** 1 Materials Innovation Area, CTAG - Automotive Technology Centre of Galicia, O Porriño, Pontevedra, 36475, Spain

**Keywords:** Recyclability, plastic, coating, automotive, electronic, tensile strength, impact strength, ABS, PC, reprocessing, mechanical performance, end-of-life

## Abstract

This research focuses on the study of the tensile modulus and impact resistance of acrylonitrile-butadiene-styrene (ABS) and its blends with polycarbonate (ABS/PC) including recycled and painted material. A comprehensive assessment was done to determine the impact of reprocessing cycles, remaining coating and their combined effect in the final properties of the recycled polymer. Post-consumer materials are in an already-aged state, lowering their initial properties. Mechanical recycling methods showed that the reprocessing cycles have a higher impact on the mechanical performance than the amount of recycling material content. Also, the material is often coated when they are about to be recycled. The remaining coating impurities play a major role in the recycling process, losing up to 42% of the impact resistance for ABS and 28% for ABS/PC. It was demonstrated that below a 10% of remaining paint, both materials retained is performance as a neat product. Impurities was declared to be the most pernicious element on the recycling process and their elimination must be a priority regarding this objective. These results provide a better knowledge of the recycling effect and can be used to decide the potential recyclability of plastic. The ascribed project of this study (DECOAT) aims to develop efficient systems to remove coatings at the end-of-life of the part, to reduce the damage and promote the use of recycled material in high-tech applications.

## Introduction

Plastics are one of the most relevant materials worldwide due to their unique combination of properties, including chemical resistance, ease of processing, low cost and lightweight potential. These properties allow them to be used in a variety of key components for different sectors, such as construction, architecture, cushioning, electronics or the aeronautical and automotive. On top of that, the increasing use of plastic materials increases the post-consumer plastic waste produced, giving concern about their management and recyclability techniques. According to a new report of the OECD organization, only about 9% of collected plastic waste is recycled while 22% is mismanaged globally. Although, recent improvements on the reusability and recyclability of plastics had been performed, the global production of plastics from recycled or secondary plastics has more than quadrupled in the last 20 years, but this is still only 6% of the size of total plastics production, being most of the plastics in use today virgin or primary plastics, made from crude oil or gas ^1^.

The automotive and electronic industries manufacture key products for the current market needs. They use high amounts of plastic materials, including polypropylene (PP), polyurethane (PU), polyvinyl chloride (PVC), acrylonitrile-butadiene-styrene (ABS), polycarbonate (PC) and many others. These sectors fulfil the demand of around a 15 % of the Europe demand for plastics materials ^2^. Among them, ABS and PC are one of the most important ones. ABS is a polymeric material that stands out for being highly resistant to impact and chemical corrosion along with a low melting point that allows an easy processability. It is widely used automotive and electronics as an impact modifier or integral part of dashboards and steering wheel covers. However, ABS has lower mechanical properties than most of the engineering plastics on the market ^3^. PC on the other hand, is a widely used amorphous plastic which has some outstanding characteristics like transparency or high impact strength but possesses poor solvent resistance and harder processability due to its high viscosity ^4^. Combining those materials in a ABS/PC blend, increases the thermomechanical resistance of ABS ^5, 6^ and reduces the cost and increases the processability of PC ^3^. ABS/PC blends are commonly applied in automotive parts as well as housings of electronics and household appliances ^7^. Automotive and electronics were one of the main demanding end-users for these kinds of plastics, accounting for about 9.9 and 6.2 % respectively of the total European plastic demand in 2018 ^8^. One of the main issues are the wastes generated from engineering plastics used in automotive and in electronic equipment, where ABS and PC take an important part. The waste generation of Waste Electrical and Electronic Equipment (WEEE) has increased from 8.3 Mt in 2010 to 10.4 Mt in 2021, whereas its collection has increased from 3.8 to 5.6 Mt ^9^. On top of that, the demand of recycled engineering plastics reached 2.8 Mt in 2020. This means that although recycling is experiencing and increasing demand, the increasing production and low collection rates show us that there is still plenty of work to do.

The most common solutions for recycling plastics include thermal, chemical or mechanical recycling. Thermal recycling recovers energy from burning plastics but this method has bad prospects in environmental aspects ^10, 11^. Chemical recycling breaks down polymers into their original building blocks by a strong degradation provoked using chemical agents. However, it has some drawbacks that come from the high environmental impact by to the use of harmful chemicals and their consideration as an expensive method ^12, 13^. On the other hand, as most of the plastics used in automotive and electronics are thermoplastics, the most recommended recycling technique is mechanical recycling. Heren, plastics are transformed into pellets and then added to the virgin plastic (or another product), which then can be used for the same or an alternative use ^14^. However, this technique cannot be used to recover thermosetting polymers (like epoxy or unsaturated polyester resins), because of their crosslinked nature ^15^.

The first step for most commercial plastics wastes is the sorting of materials into different components ^16^. After sorting, sometimes the waste quality is not enough to justify a reprocessing step and so, reusing them into solid particles for bitumen, concrete or asphalt would be a cheap and effective solution ^14^. Other reported uses included their use as reducing agents or binders ^17, 18^ as well as designed blends where the quality is good enough but enhancing circularity ^19^. In the case of blends, their processability is enhanced and properties are more versatile, problems of chemical incompatibility and of physical in- homogeneity of different plastics may arise. Brittleness, discoloration or yellowness, loss of optical clarity or degradation of mechanical properties are common issues ^15^.

Although ABS itself can be recycled through thermal or mechanical methods ^20^, for WEEE materials, solvents are commonly needed for a chemical recycling ^21^. In the automotive and electronic sectors, Plastic components are employed in visible applications, where design plays an important factor in the consumer’s decision and for that, they are commonly coated with paints or varnishes that improve their appearance and their resistance to external agents. Coatings are an additional stone on the recyclability path, as they are designed to be strongly attached to the substrate surface and resist aggressive conditions ^22, 23^. To allow its reuse, the coating must be separated from the component, adding an additional cost than just a mere mechanical recycling. However, it is very hard to eliminate all the coating impurities before the recycling process. Several studies showed that impurities decrease the mechanical properties of the recycled materials, preventing its use on demanding applications ^24– 27^. This could have implications for the industry, due to the lack of repeatability from lot to lot with recycled polymers, which would result in injury or warranty issues for the company, leading to money loss. For that reason, the highly technological sectors, such as the automotive industry, have been reluctant to use large amounts of recycled plastic, fearing a significant reduction of the material performance and thus, making, painted parts virtually not recyclable. Indeed, recycling can affect the visual appearance of the resultant material, giving additional problems ^28^. It was reported that even 1 wt.% of a polymeric impurity in composites, causes unacceptable reductions in impact resistance and ductility ^29^. Such a challenge is of industrial importance and could lead to massive improvement in the use of recyclable materials in the automotive and electronic sectors. Besides impurities, several factors can still affect the final properties of the recycled materials. The ageing of the material at the end-of-life, the number of reprocessing cycles or the amount of recycled material can all negatively impact the performances of the material ^23, 25, 30– 33^.

In the present study, a comprehensive assessment was done to evaluate the effect of each of those factors (ageing, reprocessing cycles and coating impurities) and a cross-combination of those which showed higher impact, to provide an insight about the factors influencing the performance of a recyclable material. The results provide a better knowledge of the recycling effect and can be used to decide the potential applications of recycled plastic. The ascribed project (DECOAT) aims to develop efficient systems to remove coatings at the end-of-life of the part, to reduce the damage and promote the use of recycled material in high-tech applications. Being part of a European project with industrial application, gives clear proof about the importance of close working environments between research entities and companies, giving results that impact on the society.

## Methods

The assessment was done for 2 industrial use-cases:

• Automotive use-case: A rear door garnish made of ABS coated with a solvent-based primer, a black paint and a clear coat provided by Maier S. Coop (Gernika, Spain).
• Electronic use-case: A socket part made of ABS/PC coated with a water-based anthracite paint provided by Panasonic (Istanbul, Turkey).

To perform the present study, the mentioned polymers were ABS Elix H605 Black (Elix polymers, Tarragona Spain) and ABS/PC Lupoy HT5007A White (LG Chem, South Korea). Polymer pellets where dried before injecting it with a WITTMANN DRYMAX E30 (Viena, Austria) and their moisture was measured with a Brabender Moisture meter AQUATRAC® - 3E (Germany). The granulator machine used was a WITTMANN G-MAX 23. The mixture of virgin and recycled pellets (after granulation) or painted pellets was done with a Vrieco-Nauta® laboratory mixer (HOSOKAWA MICRON B.V., Doetinchem, Netherlands)

Processing temperatures were determined according to the technical data sheet (TDS) of the selected polymers (220–240°C for ABS and 240–270°C for ABS/PC) with a mold temperature of 70°C for both cases. The studied cases were molded using an injection molding machine Engel Victory 350Tn (ENGEL, Austria), to obtain 3 kinds of samples according to the testing needs: Tensile strength (ISO-527-2 type A), impact strength (ISO-179-2) and plates of 110×160×3 mm. Average value and standard deviation of 5 samples were reported. When required, the samples were coated with their corresponding paint, according to the supplier. The injection parameters were as follows:

- For ABS, a temperature of 220–240°C for the plastificator part and 70°C for the mold. Pressure was 65–70/25–30/7 Kg/cm ^2^ (injection/post-pressure/counterpressure). The total time for injection (including all steps) was around 60 s.
- For ABS/PC, a temperature of 240–270°C for the plastificator part and 70°C for the mold. The injection machine conditions were 60–65/20–25/7 Kg/cm ^2^ (injection/post-pressure/counterpressure). The total time for injection (including all steps) was around 50 s.

Due to the requirements of this project, a single-screw injection machine was used. However, it must be punctuated that a single-screw extruder should not be used to melt-blend components, as it lacks sufficient shear forces for proper mixing. On the other hand, it is known that twin-screw cause more thermomechanical degradation.

The study was divided into two different methods, focusing on the impact of the ageing, reprocessing cycles, recycle content and amount of coating impurities on the properties of the case materials.

### 1.1. Method to assess the properties of the material at the end-of-life: effect of the ageing

**Figure 1.**
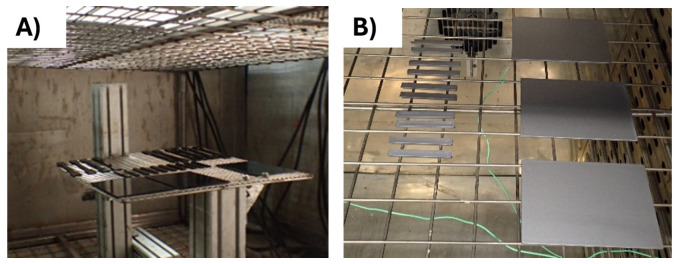
**A**) ABS samples into the climate chamber **B**) ABS/PC samples into the oven.

In a real-life scenario, the parts presumed to be recycled are already damaged due to the exposure to different environmental conditions (temperature, moisture, sun irradiation, etc.) during several years along its lifetime. For that reason, the material is already in an aged state before the start of the recycling process. Commonly, an aged material has presumably lower mechanical properties than the pristine one ^30, 31^. To evaluate this loss of properties, ABS and ABS/PC samples were exposed to accelerated ageing tests ( Figure 1), following the standard of the automotive and the electronic sector, respectively. After the ageing, the samples were tested to determine the tensile modulus and the impact resistance of the material, respectively. The use cases are depicted below:

- For the automotive use-case, DIN 75220 standard for exterior parts was followed, outlining the process of simulating prolonged solar radiation exposure. The tests consist of alternating 10 dry cycles (including irradiation) with 5 humid cycles, for a total of 15 days of accelerated ageing. The exposure was done in a climatic chamber FITOTERM 14.000 ( Figure 1A).
- For the electronic use-case, samples were exposed to 70 °C during 168 h in an oven ( Figure 1B), following an internal standard from Panasonic.

### 1.2. Method to determine the effect of the recycling process on plastic material

During the recycling process of coated parts proposed in this study, we identified different factors that can affect the properties of the material. Table 1 summarize the parameters tested for each factor. For each factor, tensile and impact strength were reported. A factorial design resume is shown in the supporting information ( **Table S1 and S2**)

**Table 1.** Design of experiment to evaluate the effect of the recycling process proposed in this study.

	FACTOR 1. Mechanical recycling	FACTOR 2. Coating impurities	FACTOR 3. Combination of factors
**Purpose**	To determine the recycled content and n° of cycles with the best compromise between environmental impact and material performance	To determine the maximum remaining coating content to guarantee the circularity and effectiveness	To determine the impact of both effects (impurities and reprocessing) on the recyclability of the material
**Variables**	- Recycled content (15%, 30%, 50%, 100%) - N° of reprocessing cycles: 1 to 5	0.5%, 1%, 3%, 10%, 25%, 50% and 100% of remaining coating respect to a fully painted part.	- 5%, 10% and 20% of coating impurities - 1–5 reprocessing cycles

￭ **The mechanical recycling process itself:**

**Figure 2.**
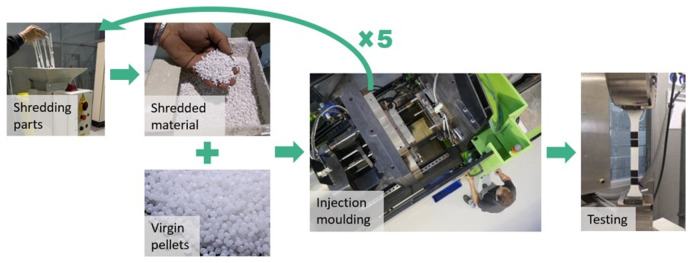
Experimental procedure to evaluate the mechanical recycling effect on the properties of plastic.

The recycling process consists of several steps of reprocessing. First, the material parts are transformed into shredded material. Then, they are mixed with virgin one at different proportions according to the desired recycled content (15, 30, 50 and 100%). Last, the material is injected to prepare the test samples according to the standards. Following this procedure, the pressure, shear, and temperature during the reprocessing can shorten the polymeric chain, affecting the mechanical properties ^22, 29^. Since a circular approach is aimed, we evaluated the properties after five reprocessing cycles and different recycled contents ( Figure 2).

￭ **The remaining coating impurities:**

**Figure 3.**
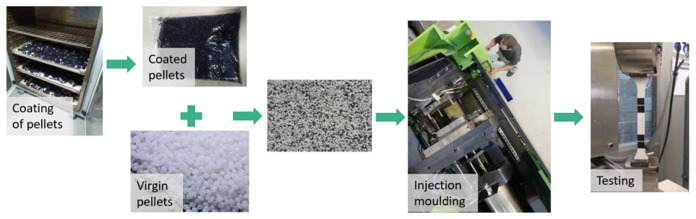
Experimental procedure to evaluate the effect of coating impurities.

Most of the recycling procedures are not perfectly efficient, therefore some coating impurities can remain in the plastic substrate, affecting its properties ^23, 25^. In this case, mostly acrylic impurities from paint residues could be expected. To reproduce this situation, plastic pellets were coated and then mixed with virgin pellets, controlling the weight of coating that was introduced in the mixture ( Figure 3). It was calculated that the automotive part (ABS) has 1,92 wt.% of coating and the electronic part (ABS/PC) has 0,40 wt.% respectively. Therefore, those proportions were considered as 100% coating impurities, corresponding to an untreated part.

￭ **Combined effect of reprocessing and coating impurities:**

Through the previous factors presented above, it was noted that the reprocessing of the material and the presence of coating impurities could have a big impact on the mechanical performance of the plastic product. For that reason, to study those combined effects, ABS and ABS/PC pellets were coated as in the process showed in Figure 3 (5, 10 and 20% of remaining coating impurities) and then subjected to a five-cycle reprocessing process. The aim of this analysis is to determine the possible synergistic effect between these recycling factors.

## Results

### Effect of the ageing on the mechanical properties of the material

Specimen samples were manufactured following the procedure explained in section 3 and then tested for tensile and impact strength. Five test samples were tested per material and their average was reported. As shown in Table 2, Aged ABS showed a significative loss of properties on both tensile modulus and impact resistance ( **Figure S1, see *Extended data***), decreasing 12.3% and 18.8% respectively in comparison to virgin ABS. On the other hand, aged ABS/PC was also damaged by such process, with a loss of properties of 4.9% and 17.0% for tensile modulus and impact resistance, respectively ( **Figure S2, see *Extended data***) in comparison to pristine ABS/PC. The change in tensile modulus for ABS/PC is not significant as showed by the statistical data showed in the supporting information document and in Table 3. ABS suffered a higher degradation on its properties than the ABS/PC blends. This is explained by the lower UV radiation resistance of ABS due to the double bonds of the butadiene unit, whereas PC is relatively stable to the light ^34^. Also, the higher tensile performance and Tg of PC, results on a higher resistance to thermal degradation than ABS, thus improving the overall properties in the blend. On top of that, it can be observed a higher damage on the impact resistance performance than in the tensile modulus in both ABS and ABS/PC. This is due to surface degradation upon ageing, mostly on the polybutadiene phase of the polymer. Ageing leads to chain scission and cross-linking, that restricts the polymer chain mobility and decreases the free volume, resulting in an increase of the Tg upon ageing, whereas the effect on the styrene-acrylonitrile phase is less significant ^31, 35^. It can be observed that automotive and electronic parts following an ageing process, suffer enough loss of mechanical properties. These results showed the importance of the ageing studies on automotive and electronic parts in the mechanical and life cycle analysis of these products.

**Table 2.** Tensile modulus and impact resistance results of test specimens after ageing tests.

Test samples	ABS-ref	ABS-After automotive ageing	ABS/PC-ref	ABS/PC-After electronic ageing
Tensile modulus (Mpa)	2407.4 ± 84.8	2110.6 ± 131.4	2276.5 ± 159.7	2165.7 ± 118.0
Impact resistance (KJ/m ^2^)	18.43 ± 0.25	14.97 ± 0.32	50.53 ± 2.00	41.93 ± 1.77

**Table 3.** ANOVA statistical studies for tensile studies.

Test performed	ANOVA test (one factor)	F value	Critical F value	P-value (<0.05)
Tensile ageing test	ABS	20.74	4.67	0.00054073
	ABS/PC	2.34	4.67	0.15004974
Tensile mechanical recycling test	ABS	2.91	3.24	0.06685515
	ABS/PC	57.85	3.24	0.00000001
Tensile modulus-Coated and reprocessed	ABS	1.73	3.89	0.21847025
	ABS/PC	0.04	3.89	0.96443924

### 1.3. Effect of the recycling and purification process on the mechanical properties

￭ Assessment of the mechanical recycling effect

**Figure 4.**
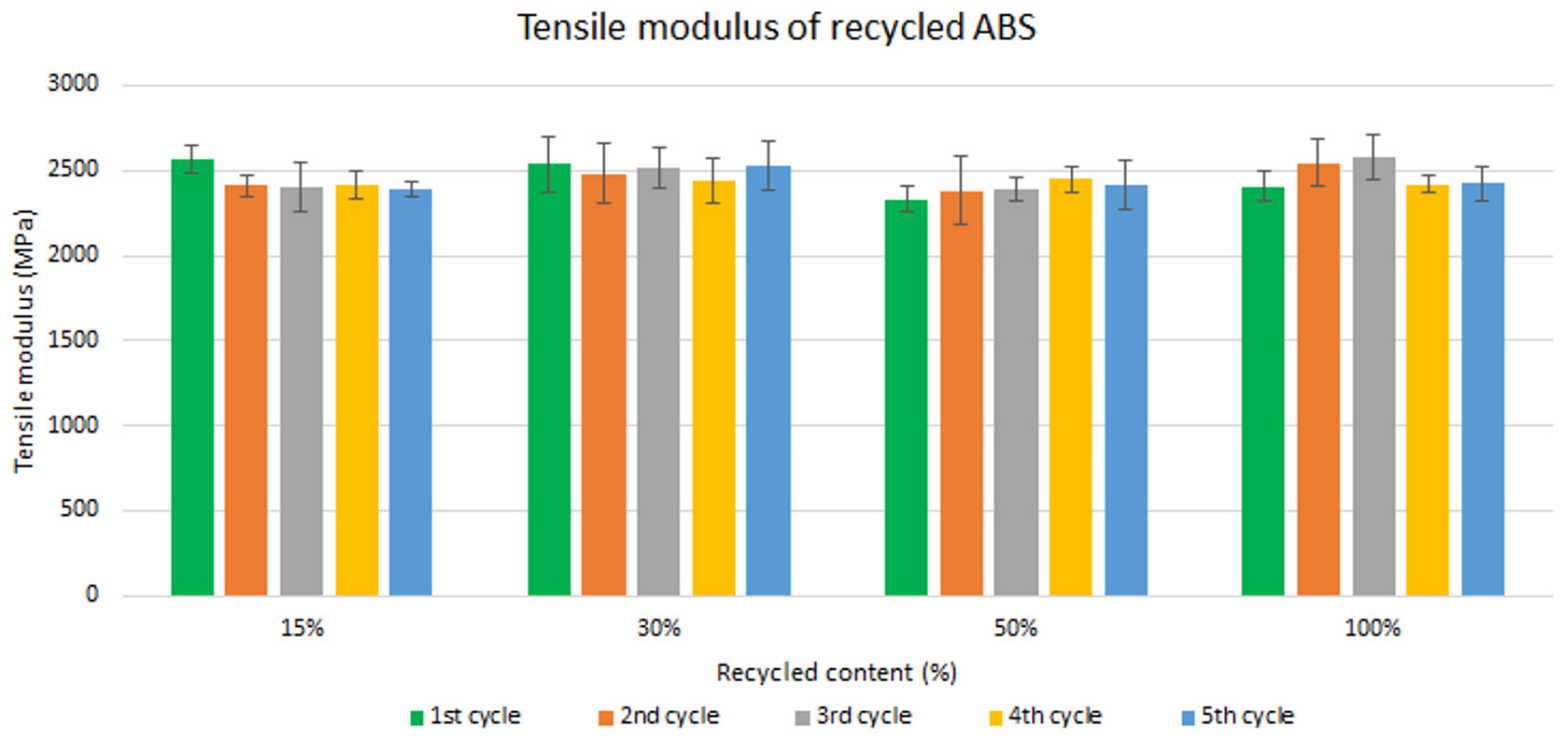
Effect of recycled content and reprocessing cycles on the tensile modulus of ABS.

**Figure 5.**
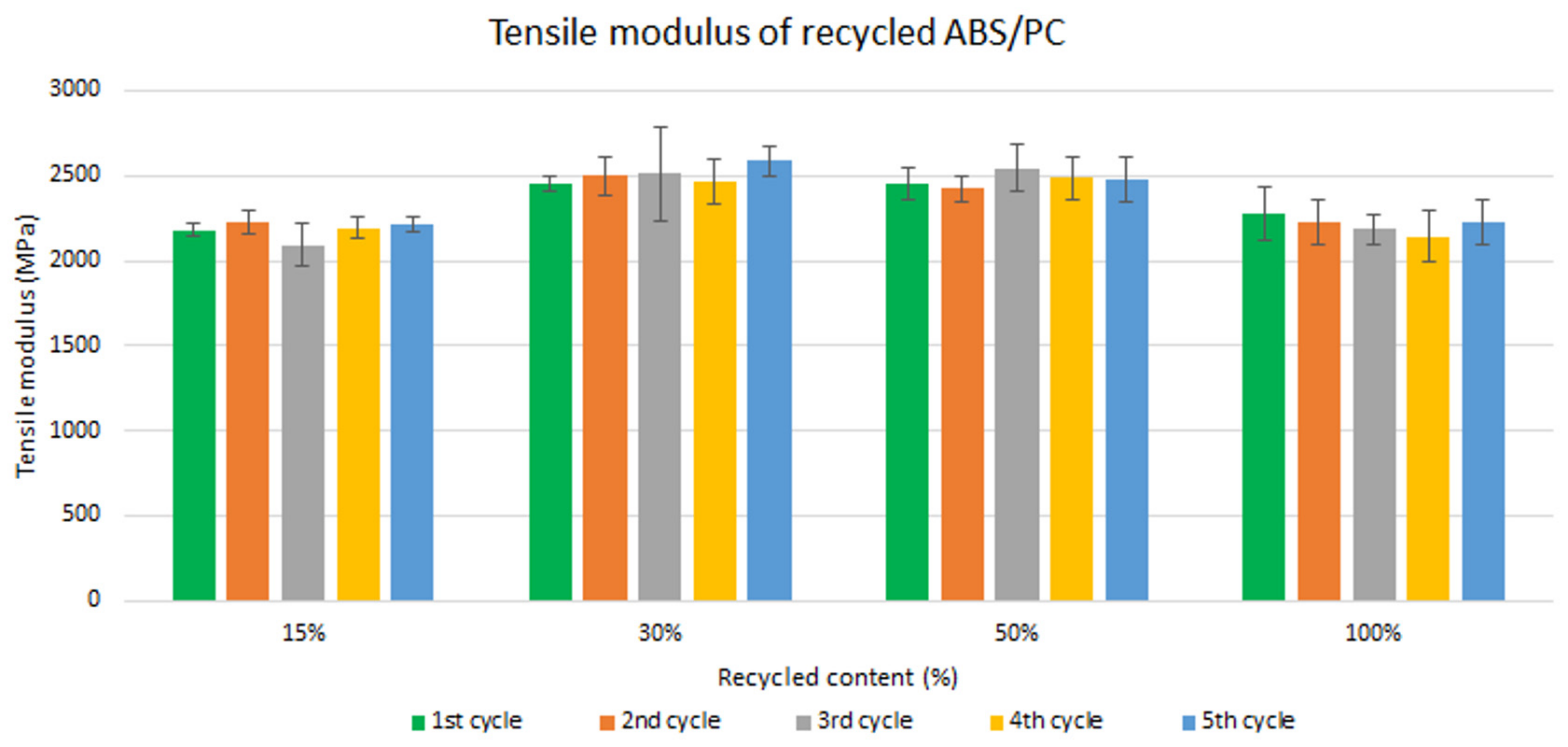
Effect of recycled content and reprocessing cycles on the tensile modulus of ABS/PC.

Samples were manufactured following the procedure explained in section 3.2 and then tested for tensile and impact strength. Five test samples were tested per material and their average was reported. Average values of tensile modulus are plotted in Figure 4 and Figure 5 for ABS and ABS/PC respectively. For recycled ABS, the tensile modulus remains very stable and close to the reference showed in Table 2 (1 ^st^ cycle of 100 % recycled material), regardless of the recycled content nor the number of reprocessing cycles ( Figure 4). These results showed the excellent behavior of ABS as a recyclable material for the automotive sector. It is not statistically significant the difference in ABS tensile modulus results, as showed in the Table 3. Regarding ABS/PC for electronics, the effect of the recycling content and different injection cycles on the tensile strength showed a positive impact ( Figure 5). Like ABS alone, the values of ABS/PC remain close to the reference one ( Table 2). Notably, an initial increase (about 10%) of the tensile modulus with the introduction of increasing content of recycled material was observed. It was reported that changes in the orientation of the polymeric chains at the macromolecular scale could lead to bear increasing loadings during the test ^36^. The reprocessing effect showed as well increasing values with a slightly higher impact than ABS alone. Degradation caused by several injections could produce a reduction in the mean distances and the length of the PC macromolecular chains, thus increasing the Van der Waals interactions and leading to an increasing tensile strength ^37^. Despite this, it is expected a reduction on the mechanical performance after more than 5 cycles due to the continuous degradative process ^38^.

**Figure 6.**
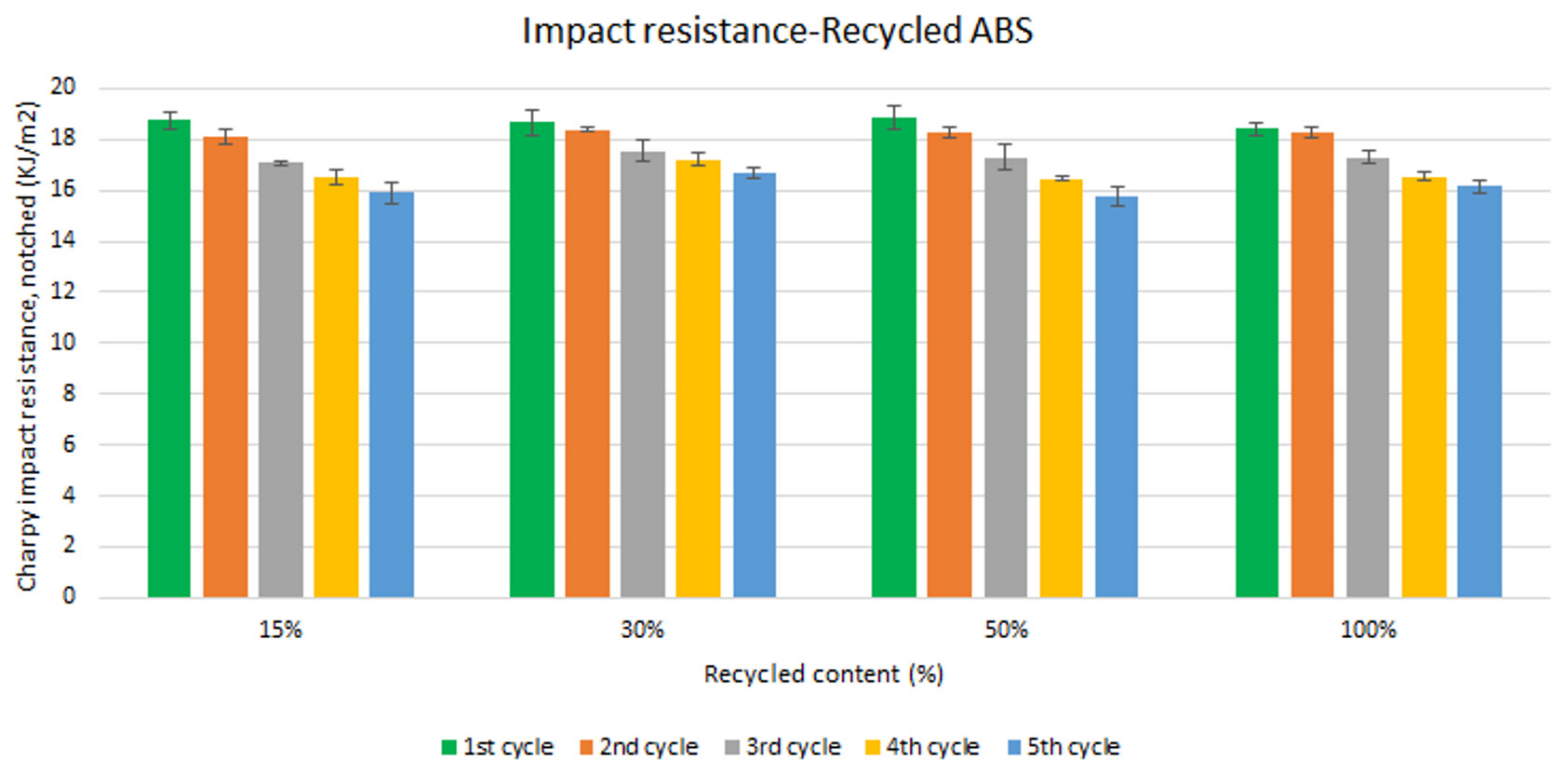
Effect of recycled content and reprocessing cycles on the impact resistance of ABS.

**Figure 7.**
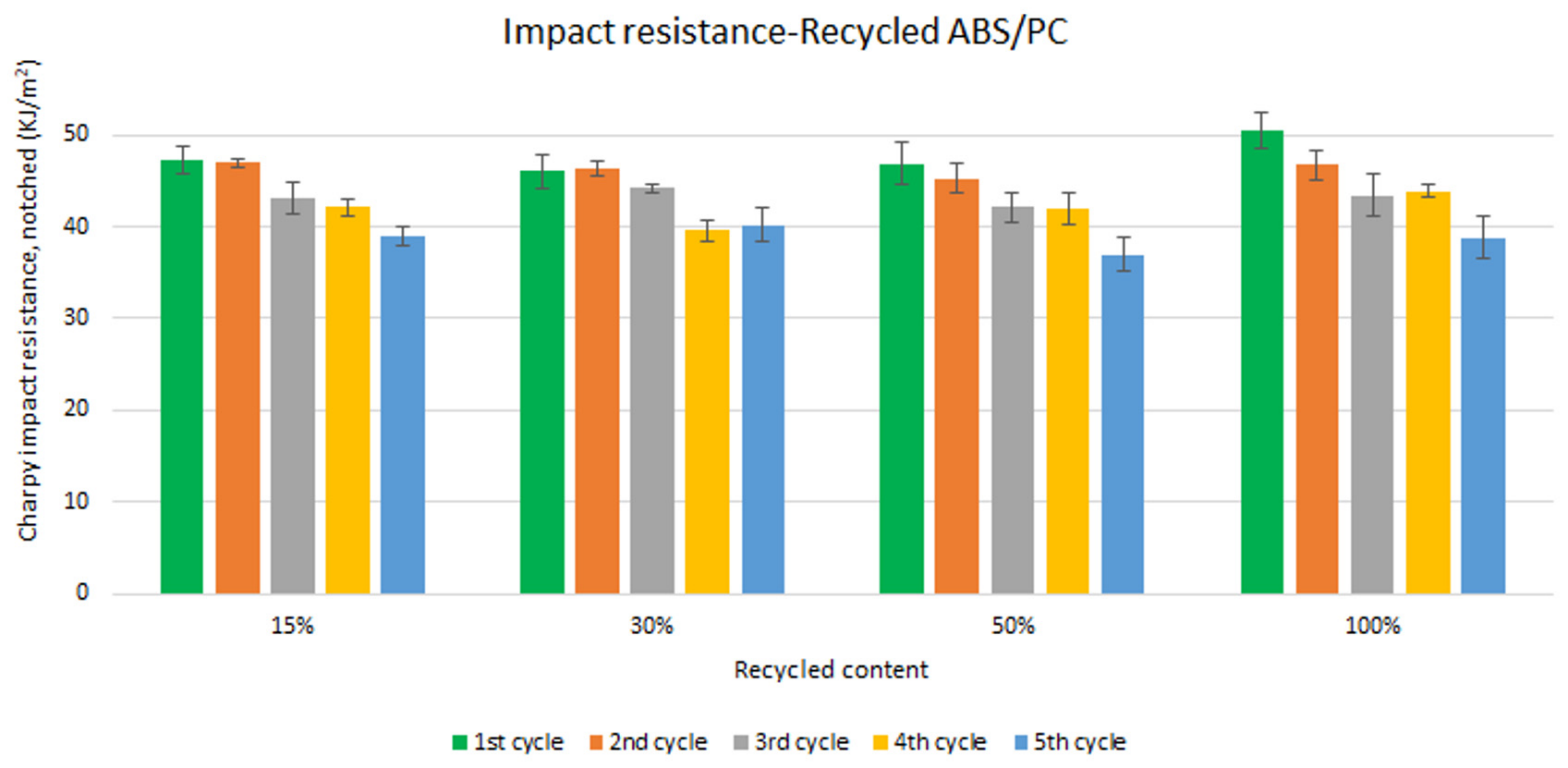
Effect of recycled content and reprocessing cycles on the impact resistance of ABS/PC.

Unlike the tensile strength, impact resistance performance changes especially with each reprocessing cycle. Five test samples were tested per material and their average was reported. Average values of charpy impact resistance are plotted in Figure 6 and Figure 7 for ABS and ABS/PC respectively. In both materials, the impact of increasing recyclable content is almost negligible, although the presence of recyclable content decreases the performance in comparison to the reference Table 2. The impact resistance dropped on average by 5.9% for ABS and 14.5% for ABS/PC when recycling content is introduced. On the other hand, the impact of the reprocessing cycles is of significance. The first cycle of each recycled content for ABS keeps similar strength as the reference ( Table 2). However, after each additional cycle, the performance drops dramatically, decreasing up to 12.5% on average for the 5 ^th^ cycle ( Figure 6). In the case of ABS/PC, the loss of performance due to the reprocessing cycles is even more dramatic than in the ABS case. ABS/PC losses impact resistance from the first reprocessing cycle and drops 23 % on average after 5 cycles ( Figure 7). On the other hand, ABS/PC seems to show higher stability on the first two reprocessing cycles regardless of the recycling content but drops abruptly after the 3 ^rd^ cycle. The loss of mechanical properties in ABS is often attributed to thermo-oxidative degradation in the polybutadiene phase only, whereas additional physical ageing can also occur in the styrene-acrylonitrile phase ^39^. The polymeric degradation of ABS is thermodynamically favorable after periods of exposure to heat and oxygen (such as after reprocessing), leading to a deterioration of the mechanical properties ^40^. Commonly, the introduction of PC leads to a higher stability upon reprocessing ^41, 42^. However, it was reported that the decrement of strength with reprocessing might be attributed to the decrease of the molecular weight and chain scission. Broader chain length distribution and shorter chain induced poor chain entanglements, and consequently, the impact strength dropped with increasing reprocessing ^3^. Further studies including thermogravimetric analysis (TGA) and differential scanning calorimetry (DSC) techniques could give more information about the degradation of the material with the increasing reprocessing cycles.

￭ Assessment of the coating impurities effect

Samples were manufactured following the procedure explained in section 3.2 ( Figure 3) and then tested for tensile and impact strength. Results are showed in Table 4, and **Figures S3** and **S4** ( **see *Extended data***). Five test samples were tested per material and their average was reported. Regarding ABS, the tensile modulus decreases when coating impurities are present. For coating contents from 0.5 to 50%, the tensile modulus decreases 12% on average. With 100% of remaining coating, it decreases 16%. For ABS/PC, the effect of coating impurities on the tensile modulus is not clear, since there is a huge variability of data which is more evident with the higher impurity content. However, a slight decrease in the performance can be presumed. For the impact resistance, again, a clear relationship was found between the amount of coating impurities and the performance of both ABS and ABS/PC. Both ABS and ABS/PC can retain its properties when the coating impurities fall below 3% compared to the neat material. However, with full coating (100% impurities) decreases the impact resistance of ABS and ABS/PC by 42% and 28%, respectively. It can be thus noted the better performance of ABS/PC with respect to ABS. According to the trend curves, it is enough with 14.2% and 21.2% of the remaining coating impurities to lost about the 10% of the performance of the reference ABS and ABS/PC, respectively. The presence of coating impurities had a very large impact on the mechanical properties. It was reported that these impurities usually have different dimensions and produce an inhomogeneous structure with weak mechanical points, which resulted in local cavitation and higher stress for the ABS matrix that allows an easy breakage ^7, 26, 43^. Further studies including scanning electron microscopy (SEM) analysis for the microstructure of the polymers would be recommendable. This result shows the importance of a good cleaning process before the start of a recycling procedure.

**Table 4.** Tensile modulus and impact resistance of ABS with varied coating impurities (%).

Material	Mechanical performance	Coating impurities (%)							
		Ref	0.50%	1.00%	3.00%	10.00%	25.00%	50.00%	100.00%
ABS	Tensile modulus (Mpa)-Avg	2407.4 ± 84.8	2129.2 ± 124.8	2120 ± 159.0	2119.8 ± 263.4	2155.6 ± 21.9	2090.1 ± 98.1	2147.1 ± 73.5	2025.8 ± 231.9
	Impact resistance (KJ/m ^2^)-Avg	18.43 ± 0.25	17.98 ± 0.18	18.53 ± 0.45	18.38 ± 1.89	17.6 ± 0.27	14.73 ± 0.4	13.13 ± 0.4	10.7 ± 0.28
ABS/PC	Tensile modulus (Mpa)-Avg	2276.5 ± 156.7	2333.5 ± 219.6	2119.5 ± 84.8	2176.4 ± 330.5	2171.9 ± 91.8	2203.3 ± 128.0	2191 ± 118.3	2431.5 ± 173.6
	Impact resistance (KJ/m ^2^)-Avg	50.53 ± 2.00	49.22 ± 2.07	49.08 ± 1.65	51.11 ± 1.73	46.09 ± 1.81	44.87 ± 1.37	41.26 ± 1.02	36.49 ± 1.11

￭ Assessment of the combined effect of coating impurities and reprocessing cycles

**Figure 8.**
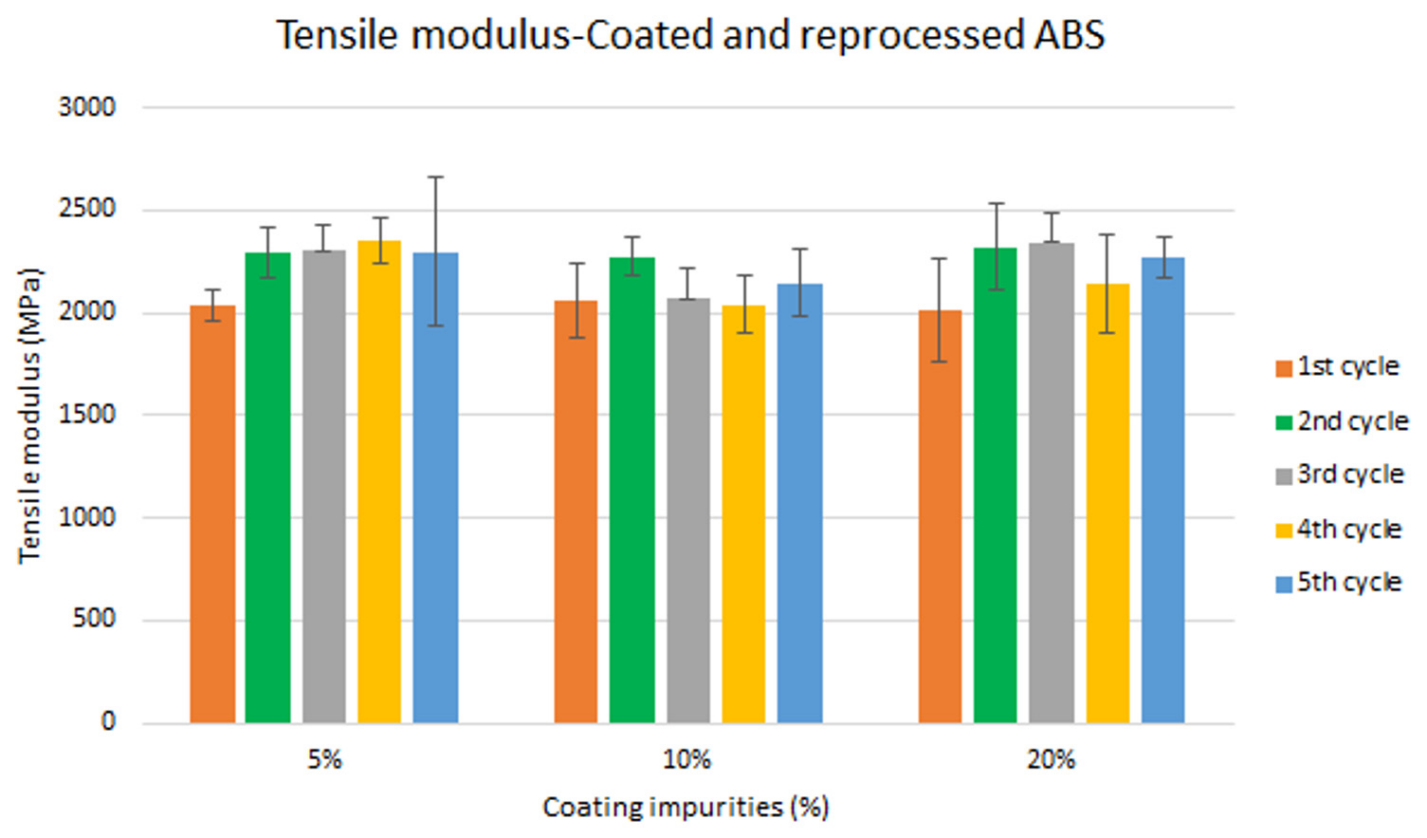
Combined effect of reprocessing cycles and coating impurities on the tensile modulus of ABS.

**Figure 9.**
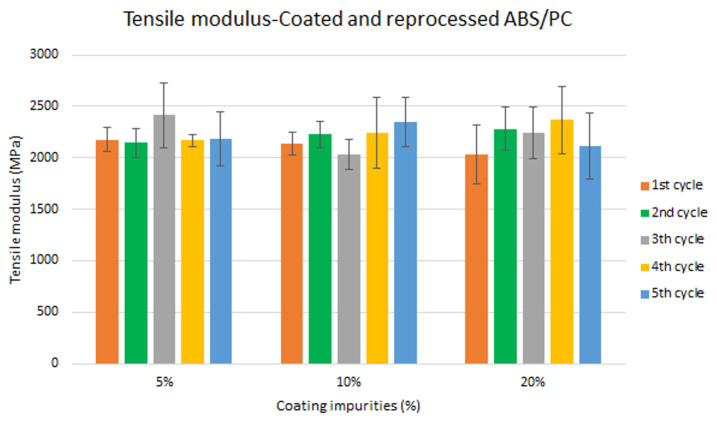
Combined effect of reprocessing cycles and coating impurities on the tensile strength of ABS/PC.

**Figure 10.**
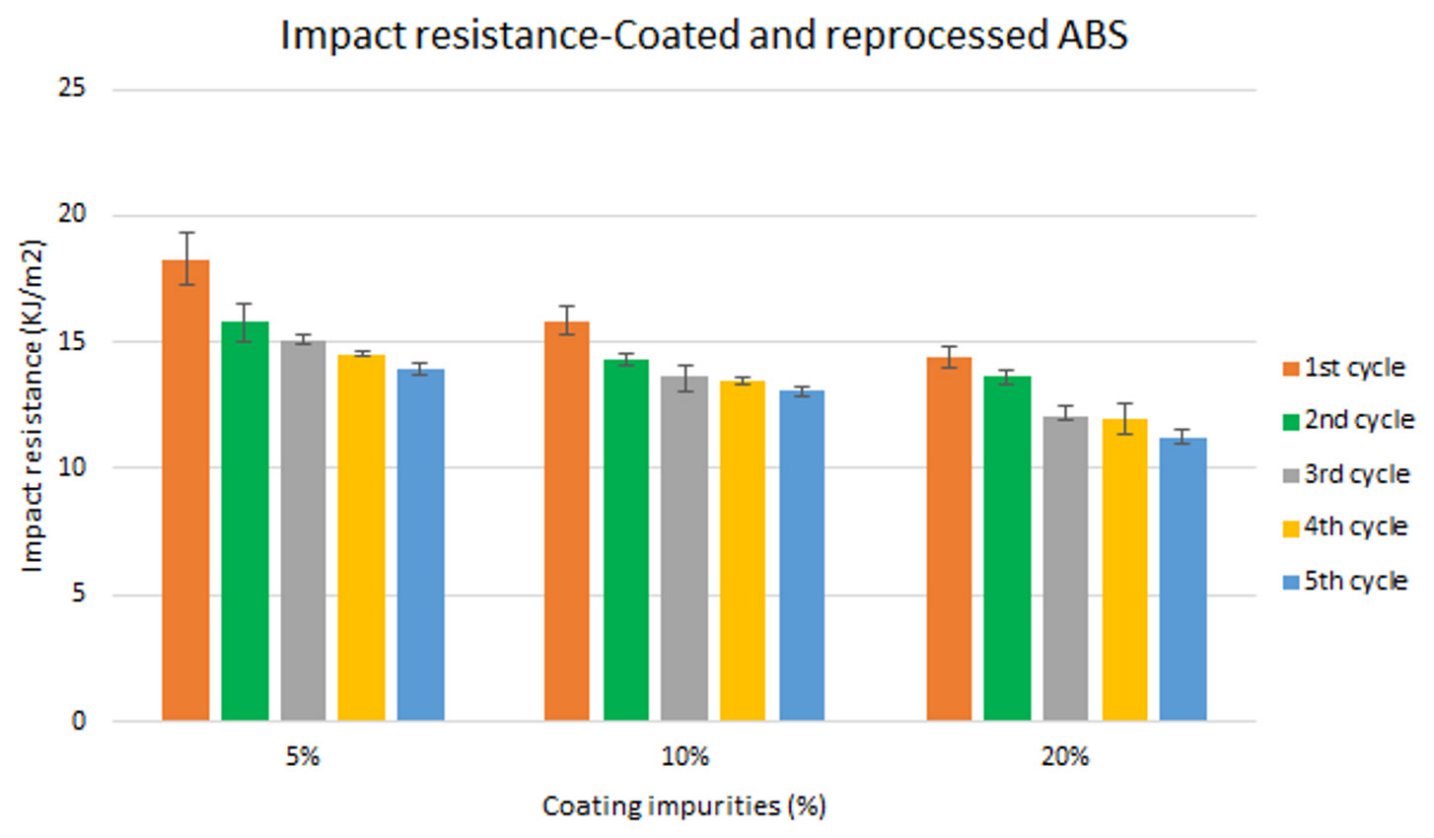
Combined effect of reprocessing cycles and coating impurities on the impact resistance of ABS.

**Figure 11.**
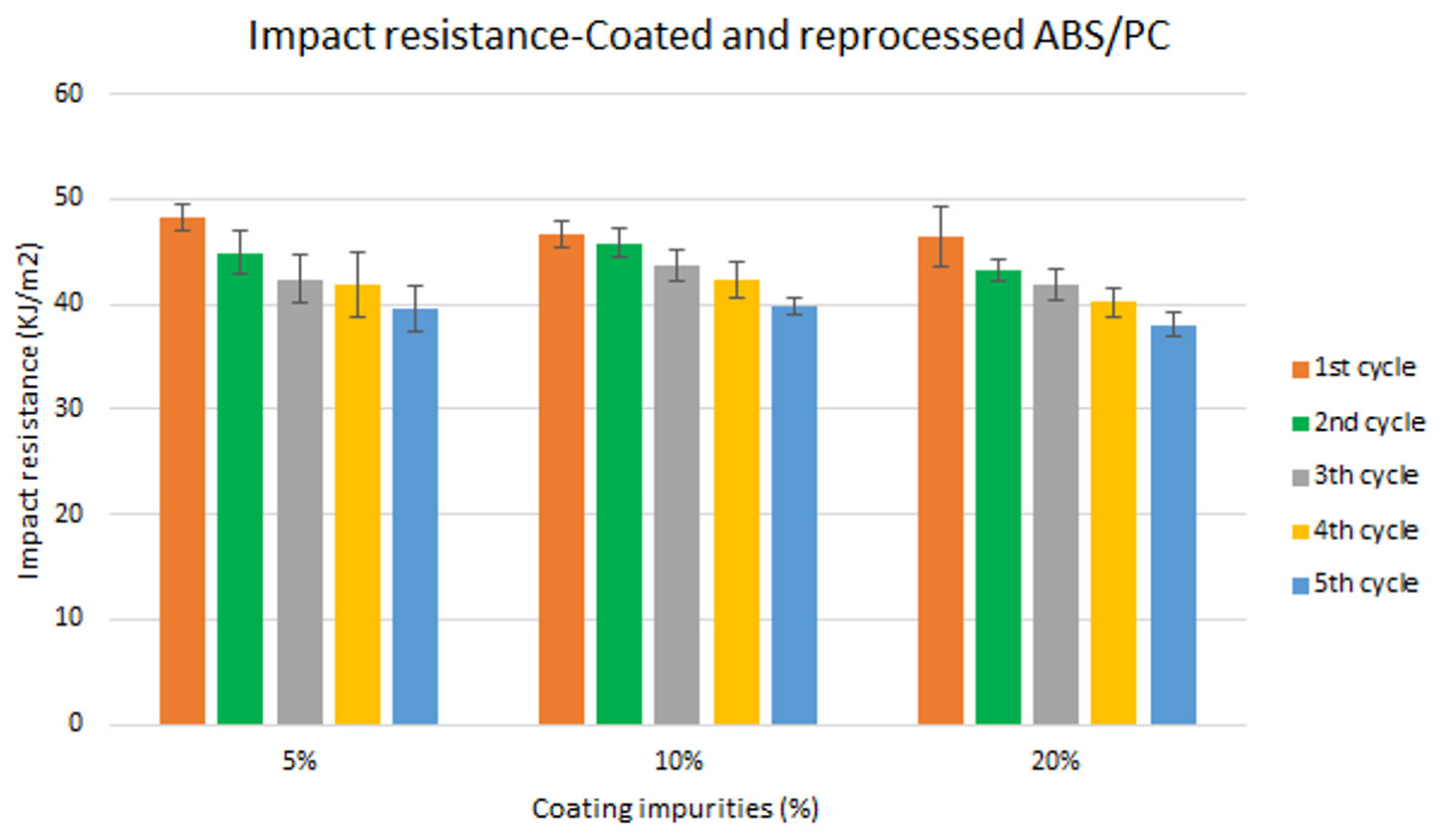
Combined effect of reprocessing cycles and coating impurities on the impact resistance of ABS/PC.

Specimen samples were manufactured following the procedure explained in section 3.2 and then tested for tensile and impact strength. Five test samples were tested per material and their average was reported. Results for ABS analysis are shown in Figure 8 and Figure 10 whereas for ABS/PC are shown in Figure 9 and Figure 11. The data for the tensile modulus and impact resistance of ABS showed a general drop on the properties of the material with increasing reprocessing cycles and coating impurities. ABS decreased on average 9% and 24% for the tensile and impact strength respectively. This result implies a higher effect on the impact resistance than in the tensile modulus. Nonetheless, the combined factors showed no synergistic effect, being the effect of the coating individually, the one with probably higher contribution. In the case of ABS/PC, the performance decreased on average by 3.0% and 15% for the tensile and impact strength respectively. ABS/PC retained notably its properties in comparison to ABS. In the same way as ABS, no synergistic effect was found. One possibility to explain this lack of synergistic degradative effects could be on the impurities size and distribution. As the materials are reprocessed, the impurities could be distributed homogeneously due to the temperature and shear stress of the process, thus reducing its size. As the impurities size diminishes, the local stress increases due to the interaction between the polymer molecules, leading to a reduced effect of coating impurities on the impact strength decrease ^43^.

• Statistical studies for tensile strength of testing materials:

An ANOVA test (one factor) was performed to check if the average values reported are statistically different from each other. Herein the statistical data for the tensile strength of ABS and ABS/PC testing probes will be shown. It can be seen that the F value is higher than their corresponding critical value in the cases of ABS for the ageing test (Figure S1) and in the ABS/PC for the mechanical recycling effect ( Figure 5). Indeed, the P-values are <0.05 in the same cases. This indicates that the averages are significantly different in these cases. In the cases where the averages are not significantly different, this could be a result of using a single screw injection machine and not using a twin screw, resulting in little chain scission.

## Conclusion

A comprehensive assessment of the mechanical properties of recycled materials was done during this, isolating different factors that can potentially damage the polymers and therefore, limit their application in high-tech sectors such as automotive and electronic industries. Through ageing tests, it was demonstrated that a part to be recycled is already damaged by years of environmental exposure. Therefore, the recycling process should have almost no impact to avoid even more degradation.

- Mechanical recycling showed that ABS and ABS/PC are stable after the first reprocessing cycle, regardless of the recycled content, but a loss of properties is reported after 2 cycles or more, affecting mostly the impact resistance.
- In coated parts, the coating impurities were proved to be very pernicious in terms of mechanical properties, especially for the impact resistance. These impurities must be eliminated allowing only almost <10 % impurities to retain the mechanical performance.
- The analysis for the combination of both previous factors showed no synergistic effect between the degradative processes. Impact resistance was the one with a high depletion on its performance.

From this study, it is encouraged by the importance of a high-performance cleaning process that avoids the presence of coating impurities in the recyclable material in order to ensure the use of recyclable products into high-end industries like automotive and electronics.

## Data Availability

The raw data cannot be made public since it is within the non-disclosure agreement (NDA) signed by the Consortium. The reader should apply for individual access to the data directly with vanesa.ventosinos@ctag.com. Data are available for readers under the terms of the Creative Commons Attribution 4.0 International license (CC-BY 4.0). [Repository]: Supporting information, images and documents: 10.5281/zenodo.15544574 This project contains the following extended data: Supporting information - Figure S1, Figure S2, Figure S3, Figure S4 and Table S1 and S2. Data are available under the terms of the Creative Commons Zero "No rights reserved" data waiver (CC0 1.0 Public domain dedication).
