# Supplementary material for: Impact of recyclability on the tensile and Impact properties of coated plastic materials for the automotive and electronic sectors
Source: Open Res Eur. 2025 Sep 9;4:51. Originally published 2024 Mar 4. [Version 4] doi: 10.12688/openreseurope.16888.4 (PMC11976218; doi:10.12688/openreseurope.16888.4)
Supplement: Peer review history

# Reviewer report: Reviewer response for version 4

**DOI:** 10.21956/openreseurope.22264.r59790

**Published:** 2026-03-24

**Authors:** Jude Shalitha Perera

**Affiliations:** 1 The University of Melbourne, Melbourne, Australia

No further comments

Is the case presented with sufficient detail to be useful for teaching or other practitioners?

Yes

Is the work clearly and accurately presented and does it cite the current literature?

Partly

If applicable, is the statistical analysis and its interpretation appropriate?

Yes

Are all the source data underlying the results available to ensure full reproducibility?

Yes

Are the conclusions drawn adequately supported by the results?

Yes

Is the background of the case’s history and progression described in sufficient detail?

Yes

Reviewer Expertise:

Consumer and kerbside plastic recycling and material recovery process.

I confirm that I have read this submission and believe that I have an appropriate level of expertise to confirm that it is of an acceptable scientific standard.

# Reviewer report: Reviewer response for version 3

**DOI:** 10.21956/openreseurope.21721.r50964

**Published:** 2025-05-21

**Authors:** Jude Shalitha Perera

**Affiliations:** 1 The University of Melbourne, Melbourne, Australia

The article presents a well-designed experimental program and results to study the effect of *pain*t coating on recycling cycles. The writing is clear and scientifically sound. However, the reviewer believes that the literature on the effects of recycling cycles is not sufficiently covered in the introduction. There is a lack of recent, relevant studies on recycling of other types of plastics, which would help provide the reader with a clearer understanding of the current state of research in this area. Also note the following corrections.

 Abstract : correct "resistance)" to "resistance"

 Introduction : 9.9 and 6.2 %, respectively

 Methods: 1. explain the acronym TDS

 2. Details regarding the tensile and impact tests are insufficient. The manuscript should specify the testing standards followed (e.g., ASTM or ISO), the sample dimensions and preparation methods, the type and model of equipment used, and the testing conditions. Providing this information is essential for the reproducibility and validation of the experimental results.

Is the case presented with sufficient detail to be useful for teaching or other practitioners?

Yes

Is the work clearly and accurately presented and does it cite the current literature?

Partly

If applicable, is the statistical analysis and its interpretation appropriate?

Yes

Are all the source data underlying the results available to ensure full reproducibility?

Yes

Are the conclusions drawn adequately supported by the results?

Yes

Is the background of the case’s history and progression described in sufficient detail?

Yes

Reviewer Expertise:

Consumer and kerbside plastic recycling and material recovery process.

I confirm that I have read this submission and believe that I have an appropriate level of expertise to confirm that it is of an acceptable scientific standard, however I have significant reservations, as outlined above.

# Response

**Published:** 2025-05-30

**Authors:** Denise GARCÍA MURIAS

- **Suggestion 1: literature expansion in introduction:**

Thanks for the suggestion. The introductory section has now included more recent literature from reviews and individual studies where it is discussed the current state of recycling techniques and their uses after recycling as well as the effects of several reprocessing (brittleness, color changes, etc.).  

- **Suggestion 2: Abstract and introduction correction:**

Thanks for the corrections. Now they have been changed in the new document version.  

- **Suggestion 3: methods. TDS explanation:**

Thanks for the suggestion. Now it has been explained in the new document version (technical data sheet).

- **Suggestion 4: Methods: Test detailed:**

Thanks for the comment. In the methods section, there were already explained the standard tests for tensile strength (ISO-527-2 type A), impact strength (ISO-179-2) and dimensions of the plates of 110×160×3 mm. Indeed, their pretreatment to avoid moisture influence was shown as well as the equipments used: “ *Polymer pellets where dried before injecting it with a WITTMANN DRYMAX E30 (Viena, Austria) and their moisture was measured with a Brabender Moisture meter AQUATRAC® - 3E (Germany)*.” Also, it was said that they were molded using an injection molding machine Engel Victory 350Tn (ENGEL, Austria) and their injection parameters. Please check the text as shown in the document: *Processing temperatures were determined according to the technical data sheet (TDS) of the selected polymers (220–240°C for ABS and 240–270°C for ABS/PC) with a mold temperature of 70ºC for both cases. The studied cases were molded using an injection molding machine Engel Victory 350Tn (ENGEL, Austria), to obtain 3 kinds of samples according to the testing needs: Tensile strength (ISO-527-2 type A), impact strength (ISO-179-2) and plates of 110×160×3 mm. Average value and standard deviation of 5 samples were reported. When required, the samples were coated with their corresponding paint, according to the supplier. The injection parameters were as follows:*

- *For ABS, a temperature of 220-240°C for the plastificator part and 70°C for the mold. Pressure was 65-70/25-30/7 Kg/cm ^2 ^(injection/post-pressure/counterpressure). The total time for injection (including all steps) was around 60 s.*
- *For ABS/PC, a temperature of 240-270°C for the plastificator part and 70°C for the mold. The injection machine conditions were 60-65/20-25/7 Kg/cm ^2 ^(injection/post-pressure/counterpressure). The total time for injection (including all steps) was around 50 s. *

*Due to the requirements of this project, a single-screw injection machine was used. However, it must be punctuated that a single-screw extruder should not be used to melt-blend components, as it lacks sufficient shear forces for proper mixing. On the other hand, it is known that twin-screw cause more thermomechanical degradation.* *The study was divided into two different methods, focusing on the impact of the ageing, reprocessing cycles, recycle content and amount of coating impurities on the properties of the case materials.*

# Reviewer report: Reviewer response for version 3

**DOI:** 10.21956/openreseurope.21721.r52571

**Published:** 2025-04-11

**Authors:** Dylan Jubinville

**Affiliations:** 1 University of Waterloo, Waterloo, ON, Canada

I would like to thank the authors for their patience with the process and for accommodating my request/attempts to enhance this manuscript.

 All the best with future work!

Is the case presented with sufficient detail to be useful for teaching or other practitioners?

Partly

Is the work clearly and accurately presented and does it cite the current literature?

Yes

If applicable, is the statistical analysis and its interpretation appropriate?

Not applicable

Are all the source data underlying the results available to ensure full reproducibility?

Yes

Are the conclusions drawn adequately supported by the results?

Partly

Is the background of the case’s history and progression described in sufficient detail?

Partly

Reviewer Expertise:

Polymer chemistry, polymer recycling, bioplastics, sustainable composites, advanced characterization, 3D printing, melt blowing, polymer and rubber foaming.

I confirm that I have read this submission and believe that I have an appropriate level of expertise to confirm that it is of an acceptable scientific standard.

# Reviewer report: Reviewer response for version 3

**DOI:** 10.21956/openreseurope.21721.r52572

**Published:** 2025-04-07

**Authors:** Peng Gao

**Affiliations:** 1 Western Washington University, University of Massachusetts Lowell, Bellingham, USA

I have checked the revision and the authors address all my comments. I think the quality of the manuscript meets the indexing  standard of the journal

Is the case presented with sufficient detail to be useful for teaching or other practitioners?

Yes

Is the work clearly and accurately presented and does it cite the current literature?

Partly

If applicable, is the statistical analysis and its interpretation appropriate?

Partly

Are all the source data underlying the results available to ensure full reproducibility?

Yes

Are the conclusions drawn adequately supported by the results?

Yes

Is the background of the case’s history and progression described in sufficient detail?

Yes

Reviewer Expertise:

Biopolymers, Injection molding, plastic sustainability, mechanical recycling, crystallization and morphology

I confirm that I have read this submission and believe that I have an appropriate level of expertise to confirm that it is of an acceptable scientific standard.

# Reviewer report: Reviewer response for version 2

**DOI:** 10.21956/openreseurope.20572.r51272

**Published:** 2025-03-04

**Authors:** Peng Gao

**Affiliations:** 1 Western Washington University, University of Massachusetts Lowell, Bellingham, USA

Please find some minor suggestions below:

 Typo: Methods: ABS/PC (LG Chem, South **Korea**)

 Methods: Did the authors dry the materials before molding? For both ABS and PC-ABS, removing moisture is critical for obtaining high mechanical performances. 

 Image quality of Figure 2 and 3 are low on my end, please double check and verify. 

 Methods: I suggest the author to include a DOE table at the end of the section. The combination of factors are unclear to me. Under FACTOR 1 described in Table 1, there are 2 individual factors have 4 and 5 different levels. Did the author perform a full-factorial design of all individual factors?

 Results: Table 2, tensile modulus is missing its unit. 

 Results: Section 1.3, I suggest the authors to present p-values or factorial plots in the main text to show factors being significant or not.

Is the case presented with sufficient detail to be useful for teaching or other practitioners?

Yes

Is the work clearly and accurately presented and does it cite the current literature?

Partly

If applicable, is the statistical analysis and its interpretation appropriate?

Partly

Are all the source data underlying the results available to ensure full reproducibility?

Yes

Are the conclusions drawn adequately supported by the results?

Yes

Is the background of the case’s history and progression described in sufficient detail?

Yes

Reviewer Expertise:

Biopolymers, Injection molding, plastic sustainability, mechanical recycling, crystallization and morphology

I confirm that I have read this submission and believe that I have an appropriate level of expertise to confirm that it is of an acceptable scientific standard, however I have significant reservations, as outlined above.

# Response

**Published:** 2025-03-26

**Authors:** Denise GARCÍA MURIAS

- **Suggestion 1: Typo (South Korea):**

Thanks for the correction. Now it has been changed in the new document version v3.  

- **Suggestion 2: Methods (Material drying):**

Thanks for the comment. It is mentioned in the document how the pellets were dried: “ *Polymer pellets where dried before injecting it with a WITTMANN DRYMAX E30 (Viena, Austria) and their moisture was measured with a Brabender Moisture meter AQUATRAC® - 3E (Germany)*.”  

- **Suggestion 3: Figure 2 and 3 quality change:**

Thanks for the comment. Figures 2 and 3 have been included from the TIFF document in the repository (10.5281/zenodo.14999538) with higher quality. We have also included the other images in a higher quality.  

- **Suggestion 4: Methods: DOE table**

Thanks for the comment. According to your suggestion, we have decided to include a DOE table in the supporting information explaining how the factors and their levels affect the material’s performance. We did not perform a full factorial design but a simple design in which we could study all the factors and their levels individually. Then, we obtained the result that coating and reprocessing could have an important effect combined. For that reason, our aim here was to evaluate a possible synergistic effect between those combined factors in one simple answer. *As it can seen in these tables, we could perform a simple evaluation of these factor to obtain an outcome that is consistent with our conclusion, which is that coating impurities have a very high impact on the final performance of recycling materials and so, it is very important to clean the materials prior to the start of the recycling process.*  

- **Suggestion 5: Table 2 missing units**

Thanks for the correction. Now it has been changed to the new document version v3.  

- **Suggestion 6: Section 1.3**

Thanks for the suggestion. It was made before an ANOVA test for all the tensile tests (see supporting information) to ensure the significance of the results. The restuls from these analysis was introduced in the novel version v3.

# Reviewer report: Reviewer response for version 2

**DOI:** 10.21956/openreseurope.20572.r50877

**Published:** 2025-02-21

**Authors:** Dylan Jubinville

**Affiliations:** 1 University of Waterloo, Waterloo, ON, Canada

I would like to thank the author(s) for completing my revisions. The revisions were well incorporated, and the comments were clear.

 However, my main concern remains largely unchanged, leaving me uncertain about how to proceed. Single-screw extruders are rarely used by plastic recyclers for mixing multiple components, though they may be used for recycling a single polymer type (e.g., upcycling).

 A single-screw extruder should not be used to melt-blend components, as it lacks sufficient shear forces for proper mixing. Without thermal r morphological analysis for correlation, I find it hard to see how the author(s) could confirm that the virgin and recycled components form a uniform when using a single screw. The authors correctly note that twin-screw extruders cause more thermomechanical degradation, but this reflects real-world conditions.

 For acceptance, the authors should include a small statement regarding the limitations of using a single-screw extruder for mixing.

Is the case presented with sufficient detail to be useful for teaching or other practitioners?

Partly

Is the work clearly and accurately presented and does it cite the current literature?

Yes

If applicable, is the statistical analysis and its interpretation appropriate?

Not applicable

Are all the source data underlying the results available to ensure full reproducibility?

Yes

Are the conclusions drawn adequately supported by the results?

Partly

Is the background of the case’s history and progression described in sufficient detail?

Partly

Reviewer Expertise:

Polymer chemistry, polymer recycling, bioplastics, sustainable composites, advanced characterization, 3D printing, melt blowing, polymer and rubber foaming.

I confirm that I have read this submission and believe that I have an appropriate level of expertise to confirm that it is of an acceptable scientific standard, however I have significant reservations, as outlined above.

# Response

**Published:** 2025-03-26

**Authors:** Denise GARCÍA MURIAS

Thanks for the valuable suggestion. A statement has been included in the methods section in page 5 in the novel version v3 to highlight the limitations of using single-screw extruders for this study.

# Reviewer report: Reviewer response for version 1

**DOI:** 10.21956/openreseurope.18250.r39863

**Published:** 2024-08-14

**Authors:** Dylan Jubinville

**Affiliations:** 1 University of Waterloo, Waterloo, ON, Canada

The work for review presents a clear and interesting approach to the global issue of waste accumulation by focusing on ABS, PC, and blends in the forms of virgin and post-consumer materials. The authors discussed the degradation of the plastic component may experience during its service life which more often results in a deterioration of results. 

 There are some grammar issues found throughout the paper but nothing too impactful that the paper's message is lost. Please ensure to give another read-through.

 Please note, that the bolded numbered parts must be addressed for the article to be scientifically sound (as required by the reviewer form).

 --Introduction --

 The introduction was written clearly and with good flow.

1. **I would suggest adding a line for the specific production/waste or collection rate for ABS and PC plastics to strengthen why these two materials should be of focus.**
2. Polymer names are common nouns, and as such should not need to be capitalized, please finish on page 3 second paragraph. The capitalization may be just for the acronym but it is still an incorrect writing technique.
3. The authors state even 1 wt.% of impurity in ABS can cause unacceptable reductions in results however, this is usually the case for most polymers. I would suggest listing specific impurities in ABS processing or leaving it more general to most plastics.
4. I would argue high technology sector's hesitation to implement recycled compounds is due to the lack of repeatability from lot to lot with recycled polymers which would result in injury or warranty issues for the company, leading to money loss. This is opposed to just the materials' performance itself just decreasing the mechanical properties.
5. **The authors write that polymers and their properties all decrease as a result of aging however this is not true. If material crosslinks and branches as a result of degradation then viscosity and tensile strength increases. If the material is annealed, then the modulus and overall hardness increases. Please correct that statement on page 3 end of paragraph 2.**

-- Materials and Methods --

1. **Did the authors dry the PC and ABS/PC blends?**
  1. **ABS may be hydrophobic but PC is reported to be broadline and other papers have reported drying PC before processing.**
2. **Do the authors have the exact formulations for the 2 industrial case studies?**
  1. **The post-consumer parts can/be filled with a ton of additives to prevent aging which will offset the trend there were seen. As well, the date or production and or length of service will also have a significant impact on the properties of the post-consumer parts.**
3. Are PC and ABS compatible with one another in blending operations?

-- Results --

 The results for this work are what would have been expected from this type of work. However, 2 major issues are present in this work.

1. **The material was seemingly "recycled" in an injection molding machine which is not an adequate method for studying this. It is not adequate because this type of recycling process (post-consumer (e.g. down cycling)) typically includes a twin screw extruder which imparts greater thermo-mechanical forces such as shear and heat compared to a single screw injection molding machine. In addition, the residence time in an extruder would be different when compared to an Injection molding machine. **
** Also, if blending the recycled offsets into a virgin matrix via injection molding cause the results, in general, to be questioned as the mixing of the different constituents would be poor and not efficient as single screw set-ups are used to convey material from one end (feeding zone) to the other end (die). They are NOT to mix/blend like a twin screw set-up would.**
  The authors need to justify this for this manuscript to be accepted.
2. **The tensile strength results are not statistically significant and with the lack of mention I would assume no ANOVA was performed on these results. However, looking at the means of the results, show that these results are highly likely to all be statistically insignificant (FIG 4, 5, 8, 9). This is not bad, necessarily, as it just means the material is more stable but the results discussion needs to reflect the no significance in tensile results.**
** Please provide an ANOVA to prove your tensile results are significant.**
  The lack of tensile results may be a result of "recycling" within a single screw and not using a twin screw resulting in little chain scission.
3. **With the last point, the ABS/PC ref to after aging electronic, I would argue is not significant. Please check with ANOVA.**
4. **To prove chain scission, molecular weight analysis through whatever means (GPC, DLS, rheology) should be included.**
5. **The authors are constantly flipping between tensile strength and modulus. I assumed this is all tensile strength but please look through the entire work and fix it.**
6. **Please include the standard deviation in Table 3.**
7. **Include 100% cycle 1 in bar graphs. The reader should not have to work to compare your results.**
8. Was the supporting information file uploaded as well?
  I could not locate it so please ensure you have uploaded it.

-- Conclusion --

 No comment

 -- Abstract -- 

 Well written and concise - no other comment.

Is the case presented with sufficient detail to be useful for teaching or other practitioners?

Partly

Is the work clearly and accurately presented and does it cite the current literature?

Yes

If applicable, is the statistical analysis and its interpretation appropriate?

Not applicable

Are all the source data underlying the results available to ensure full reproducibility?

Yes

Are the conclusions drawn adequately supported by the results?

Partly

Is the background of the case’s history and progression described in sufficient detail?

Partly

Reviewer Expertise:

Polymeric recycling, processing/compounding of blends and composites as well as polymer characterization (advanced/standard).

I confirm that I have read this submission and believe that I have an appropriate level of expertise to confirm that it is of an acceptable scientific standard, however I have significant reservations, as outlined above.

# Response

**Published:** 2024-11-22

**Authors:** Denise GARCÍA MURIAS

--Introduction --

1. **I would suggest adding a line for the specific production/waste or collection rate for ABS and PC plastics to strengthen why these two materials should be of focus.**

**ANSWER: **A paragraph has been included in the next version of the article submitted, within the introduction section, highlighting the waste and collection rates of these polymers.

1. Polymer names are common nouns, and as such should not need to be capitalized, please finish on page 3 second paragraph. The capitalization may be just for the acronym but it is still an incorrect writing technique.

**ANSWER: **Thanks for the comments, polymers nouns have been fixed accordingly (mainly found in the introduction) in the next version submitted.

1. The authors state even 1 wt.% of impurity in ABS can cause unacceptable reductions in results however, this is usually the case for most polymers. I would suggest listing specific impurities in ABS processing or leaving it more general to most plastics.

**ANSWER: **Thanks for the valuable suggestion. The reference was adjusted to show a more general view of the phenomena.

1. I would argue high technology sector's hesitation to implement recycled compounds is due to the lack of repeatability from lot to lot with recycled polymers which would result in injury or warranty issues for the company, leading to money loss. This is opposed to just the materials' performance itself just decreasing the mechanical properties.

**ANSWER: **Thanks for the suggestion, we introduced a comment highlighting this possible fact in the introduction.

1. **The authors write that polymers and their properties all decrease as a result of aging however this is not true. If material crosslinks and branches as a result of degradation then viscosity and tensile strength increases. If the material is annealed, then the modulus and overall hardness increases. Please correct that statement on page 3 end of paragraph 2.**

**ANSWER: **Thanks for the comment. We stated there are several factors that can affect the performance of the material at the EOL, ageing, impurities presence, reprocessing cycles and amount of recycled material. It does not mean they decrease; it means they “impact negatively” on the performance of the material where the modified properties resultant after they EOL could be probably not desired. -- Materials and Methods --

1. **Did the authors dry the PC and ABS/PC blends?**
  1. **ABS may be hydrophobic but PC is reported to be broadline and other papers have reported drying PC before processing.**

**ANSWER: **Yes, the materials were dried before the injection procedure. Polymers where dried before injecting it with a WITTMANN DRYMAX E30and their moisture was measured with a Brabender Moisture meter AQUATRAC® - 3E. This comment was included in the methods section.

1. **Do the authors have the exact formulations for the 2 industrial case studies?**
  1. **The post-consumer parts can/be filled with a ton of additives to prevent aging which will offset the trend there were seen. As well, the date or production and or length of service will also have a significant impact on the properties of the post-consumer parts.**

**ANSWER: **Thanks for the suggestion. Unfortunately, the exact formulations cannot be disclosure as they are commercial products belonging to a private consortium in a European project.

1. Are PC and ABS compatible with one another in blending operations?

**ANSWER: **Thanks for the question. For this project, the PC/ABS was selected from the commercially ones available in the market. In this case, a (PC + ABS) Lupoy HR5007A White specially used for injection molding was chosen. -- Results --

 The results for this work are what would have been expected from this type of work. However, 2 major issues are present in this work.

1. **The material was seemingly "recycled" in an injection molding machine which is not an adequate method for studying this. It is not adequate because this type of recycling process (post-consumer (e.g. down cycling)) typically includes a twin screw extruder which imparts greater thermo-mechanical forces such as shear and heat compared to a single screw injection molding machine. In addition, the residence time in an extruder would be different when compared to an Injection molding machine. **
** Also, if blending the recycled offsets into a virgin matrix via injection molding cause the results, in general, to be questioned as the mixing of the different constituents would be poor and not efficient as single screw set-ups are used to convey material from one end (feeding zone) to the other end (die). They are NOT to mix/blend like a twin-screw set-up would.**
  The authors need to justify this for this manuscript to be accepted. 

**ANSWER: **Thanks for the question and explanation. We think that the reviewer is mostly right about his/her assumption. Our machine is an injection molding machine Engel Victory 350Tn. It consists of a single-screw injection molding machine. The procedure for testing the mechanical recycling effect was as follows, first, the material (recycled) was shredded into new small pieces and then mixed with virgin ones in a mixing machine. Then, they are injected again to manufacture new tests samples. We used this procedure as it was the common one for the recycling industry, being our intention to be a real simulation of the loss of properties only during this step. Although by using and extruder machine the mixing would be better, we did not use an extruder machine as we believe that this could bring additional thermo-mechanical stress to the material, making the comparison to the virgin material even harder and not aligned within the aim of the project. We introduced comments in the method section including the mixing machine we were using to obtain the pellets for injection.

1. **The tensile strength results are not statistically significant and with the lack of mention I would assume no ANOVA was performed on these results. However, looking at the means of the results, show that these results are highly likely to all be statistically insignificant (FIG 4, 5, 8, 9). This is not bad, necessarily, as it just means the material is more stable but the results discussion needs to reflect the no significance in tensile results.**
** Please provide an ANOVA to prove your tensile results are significant.**
  The lack of tensile results may be a result of "recycling" within a single screw and not using a twin screw resulting in little chain scission.

**ANSWER: **Thanks a lot for the suggestions. We performed ANOVA (one factor variance) in the results from figures 4,5,8 and 9. The data from Figure 4 showed that there is not a significant variance on the results: ANÁLISIS DE VARIANZA *Origen de las variaciones* *Suma de cuadrados* *Grados de libertad* *Promedio de los cuadrados* *F* *Probabilidad* *Valor crítico para F* Entre grupos 33449.55 3 11149.85 **2.906305** **0.0668551** **3.238872** Dentro de los grupos 61382.96 16 3836.435                     Total 94832.51 19         The data from Figure 5 showed that there is a significant variance on the results: ANÁLISIS DE VARIANZA *Origen de las variaciones* *Suma de cuadrados* *Grados de libertad* *Promedio de los cuadrados* ***F*** ***Probabilidad*** ***Valor crítico para F*** Entre grupos 439245.7 3 146415.2 **57.84654** **8.28E-09** **3.238872** Dentro de los grupos 40497.56 16 2531.098                     Total 479743.3 19         For Figure 8, results showed that there is not a significant variance: ANÁLISIS DE VARIANZA *Origen de las variaciones* *Suma de cuadrados* *Grados de libertad* *Promedio de los cuadrados* ***F*** ***Probabilidad*** ***Valor crítico para F*** Entre grupos 50705.5281 2 25352.7641 **1.73130012** **0.21847025** **3.88529383** Dentro de los grupos 175725.263 12 14643.7719                     Total 226430.791 14         For Figure 8, results showed that there is not a significant variance: ANÁLISIS DE VARIANZA *Origen de las variaciones* *Suma de cuadrados* *Grados de libertad* *Promedio de los cuadrados* *F* *Probabilidad* *Valor crítico para F* Entre grupos 1082.1056 2 541.0528 0.03631791 0.96443924 3.88529383 Dentro de los grupos 178772.201 12 14897.6834                     Total 179854.307 14         Based on this, a comment was included in the discussion showing this results and a sum-up table was included in the supporting information along with a brief discussion.

1. **With the last point, the ABS/PC ref to after aging electronic, I would argue is not significant. Please check with ANOVA.**

**ANSWER: **Thanks for the suggestion. We checked by analysis of variance (ANOVA) at 95% confidence level the results and found that whereas ABS showed important differences, ABS/PC performed in a more resilient way. ANÁLISIS DE VARIANZA-ABS tensile test *Origen de las variaciones* *Suma de cuadrados* *Grados de libertad* *Promedio de los cuadrados* *F* *Probabilidad* *Valor crítico para F* Entre grupos 293697.4541 1 293697.454 20.7431082 0.00054073 4.66719273 Dentro de los grupos 184064.3587 13 14158.7968                     Total 477761.8128 14           ANÁLISIS DE VARIANZA-ABS/PC tensile test *Origen de las variaciones* *Suma de cuadrados* *Grados de libertad* *Promedio de los cuadrados* *F* *Probabilidad* *Valor crítico para F* Entre grupos 40924.3494 1 40924.3494 2.3400345 0.15004974 4.66719273 Dentro de los grupos 227354.144 13 17488.7803                     Total 268278.494 14         As you can check above, the results for ABS are far more divergent than for ABS/PC. A comment was included in the “Effect of the ageing on the mechanical properties of the material” results part.

1. **To prove chain scission, molecular weight analysis through whatever means (GPC, DLS, rheology) should be included.**

**ANSWER: **Thanks for the suggestion. We agree that these tests could help to obtain better insights into the results. Unfortunately, these tests were not included into project proposal, and we do not have such techniques in our facilities portfolio.

1. **The authors are constantly flipping between tensile strength and modulus. I assumed this is all tensile strength but please look through the entire work and fix it.**

**ANSWER: **Thanks for pointing out this fact. We found that it is better to harmonize everything on “tensile modulus” and “impact resistance”. Following this, a revision was made through all the document and all images have been modified accordingly. **Please include the standard deviation in Table 3.**

**ANSWER: **Thanks for the suggestion. Table 3 was modified to include the standard deviation.

1. **Include 100% cycle 1 in bar graphs. The reader should not have to work to compare your results.**

**ANSWER: **Thanks for the valuable suggestion, Figures from 4 to 7 has been modified according to your comments.

1. Was the supporting information file uploaded as well?
  I could not locate it so please ensure you have uploaded it.

**ANSWER: **Yes, supporting information document was also included in the next version submitted. -- Conclusion --

 No comment

# Reviewer report: Reviewer response for version 1

**DOI:** 10.21956/openreseurope.18250.r40703

**Published:** 2024-06-10

**Authors:** Macaulay Mfon Owen

**Affiliations:** 1 Universiti Tenaga Nasional, Kajang, Selangor, Malaysia

**Reviewer's comments/ Reports:**

 The article titled "Impact of recyclability on the mechanical properties of coated plastic materials for the automotive and electronic sectors" have been reviewed.

 The research focuses on the study of the mechanical properties (tensile and impact strength) of Acrylonitrile-Butadiene-Styrene (ABS) and its blends with Polycarbonate (ABS/PC) including recycled and painted material, to determine the impact of ageing, reprocessing cycles, and coating impurities on the final properties of the recycled polymer.

 The research is an interesting study, and well written within the scope. However, a significant revision is needed to further improve on the quality of the manuscript.

 The research novelty must be clearly emphasized with strong justifications in the introduction and literature sections, as there are lot of work already done on ABS, blends of ABS/PC and their recyclability.

**Research title:**

 I am of the opinion that the title should be reviewed as *" **Impact of recyclability on the tensile and Impact properties of coated plastic materials for the automotive and electronic sectors*** "

 Because only tensile and impact tests were done.

**Methods:**

 State the processing temperature, pressure and time used.

 How did you determine the processing temperature used for the injection moulding of the recycled ABS, and ABS/PC?.

**Mechanical recycling process:**

 Mention the equipment used for the shredding process.

**Coating Impurities:**

 Explain the coating impurities and give some examples.

 List out the parameters of the five-cycle reprocessing process used.

**Characterizations and Testing**

**Mechanical tests; **Why did you not conduct other mechanical tests such as compressive, flexural, hardness, etc.,

**Thermal Analysis;**

 There are no any thermal analysis mentioned in your work. Why did you not conduct DSC and TGA?

**Microstructural Analysis;**

 Why didn't you carry out Scanning Electron Microscope SEM?

Is the case presented with sufficient detail to be useful for teaching or other practitioners?

Yes

Is the work clearly and accurately presented and does it cite the current literature?

Partly

If applicable, is the statistical analysis and its interpretation appropriate?

Yes

Are all the source data underlying the results available to ensure full reproducibility?

No

Are the conclusions drawn adequately supported by the results?

Partly

Is the background of the case’s history and progression described in sufficient detail?

Yes

Reviewer Expertise:

Engineering thermoplastic polymer processing, Polymer composites, Fiber reinforced composites, Materials processing, characterization and testing, Textiles, Fibers, wovens and Nonwovens, Chemical treatments,

I confirm that I have read this submission and believe that I have an appropriate level of expertise to confirm that it is of an acceptable scientific standard, however I have significant reservations, as outlined above.

# Response

**Published:** 2024-11-22

**Authors:** Denise GARCÍA MURIAS

**Reviewer's comments/ Reports +** **Replies from authors in blue below:**

 The article titled "Impact of recyclability on the mechanical properties of coated plastic materials for the automotive and electronic sectors" have been reviewed.

 The research focuses on the study of the mechanical properties (tensile and impact strength) of Acrylonitrile-Butadiene-Styrene (ABS) and its blends with Polycarbonate (ABS/PC) including recycled and painted material, to determine the impact of ageing, reprocessing cycles, and coating impurities on the final properties of the recycled polymer.

 The research is an interesting study, and well written within the scope. However, a significant revision is needed to further improve on the quality of the manuscript.

 The research novelty must be clearly emphasized with strong justifications in the introduction and literature sections, as there are lot of work already done on ABS, blends of ABS/PC and their recyclability. Within the next version of the article submitted today, a comment was included in the introduction highlighting the differentiation between this study as a proof of closed working and the common research studies.  

- **Suggestion 1: Research title:**

*I am of the opinion that the title should be reviewed as "Impact of recyclability on the tensile and Impact properties of coated plastic materials for the automotive and electronic sectors" Because only tensile and impact tests were done.*

**Answer 1:**

 Thanks for the suggestion, we changed the title accordingly in the next version submitted as we believe it matches better the content.  

- **Suggestion 2: Methods:**

*State the processing temperature, pressure and time used.* *How did you determine the processing temperature used for the injection moulding of the recycled ABS, and ABS/PC?.* **Answer 2:**

 Thanks for the valuable suggestion. The processing temperatures were determined according to the TDS of the selected polymers:

- For ABS, a temperature of 220-240 ºC for the plastificator part and 70 ºC for the mold. Pressure was 65-70/25-30/7 Kg/cm ^2 ^(injection/post-pressure/counterpressure). The total time for injection (including all steps) was around 60 s.
- For ABS/PC, a temperature of 240-270 ºC for the plastificator part and 70 ºC for the mold. The injection machine conditions were 60-65/20-25/7 Kg/cm ^2 ^(injection/post-pressure/counterpressure). The total time for injection (including all steps) was around 50 s.

A comment stating this parameters are now included in the methods section.  

- **Suggestion 3: Mechanical recycling process:**

*Mention the equipment used for the shredding process.*

**Answer 3:** Thanks for the comment. The granulator machine used was a WITTMANN G-MAX 23. This was included in the draft in in the methods section.  

- **Suggestion 4: Coating Impurities:**

*Explain the coating impurities and give some examples.*

*List out the parameters of the five-cycle reprocessing process used.*

**Answer 4:** Thanks for the suggestion and focus on injection parameters. We believe it could improve our article. The coating impurities were made mostly of paint residues. According to the TDS for the coatings, these are made of an acrylic-based coating. A comment highlighting this was included in page 7. The conditions for the five cycles were similar as the ones stated before in **Answer 2**. Temperatures were decreased a bit whereas slightly pressures were augmented to increase fluidity and compactness of the resulting testing pieces.

**Characterizations and Testing**

- **Suggestion 5: Mechanical tests:**

*Why did you not conduct other mechanical tests such as compressive, flexural, hardness, etc.,*

**Answer 5:** Thanks for the tests suggestions. Other mechanical tests were not included as they were not part of the project nor included into the assigned budget. Tensile and impact were chosen as the ones with higher impact and representativity for the study. Although we believe that if the study was more general with regard to the mechanical properties, the mentioned tests could be a good addition.  

- **Suggestion 6: Thermal Analysis:**

*There are no any thermal analysis mentioned in your work. Why did you not conduct DSC and TGA?*

**Answer 6:** Thanks for the suggestions. We believe that these techniques could bring higher insights about the degradation of the polymeric chains due to thermomechanical stress. However, in a similar way as the last suggestion, DSC and TGA were not included in the project, and it is very hard to perform them now as they are out of the assigned budget or tasks included into our work packages. Nonetheless, the objective of the article is to study the impact of recyclability on the mechanical properties, although we are aware of the relation with thermal processes. As it could be necessary to include newer tests on the study, some comments about the needs of future tests for SEM were included into the draft in page 12.  

- **Suggestion 7: Microstructural Analysis:**

*Why didn't you carry out Scanning Electron Microscope SEM?*

**Answer 7:** Thanks for the valuable suggestions. SEM is a very interesting technique to include that could give us deep knowledge about the microstructure of the polymers. Unfortunately, we do not have such technique into our facilities and this test was not included as part of the budget for the related European project. As there was a focus on newer tests to include more information than the current one, some comments about the needs of future tests for SEM were included into the draft in page 13.
